# Examining the mediating role of motivation in the relationships between teacher-created motivational climates and quality of engagement in secondary school physical education

**DOI:** 10.1371/journal.pone.0316729

**Published:** 2025-01-30

**Authors:** Daniel Milton, Paul R. Appleton, Eleanor Quested, Anna Bryant, Joan L. Duda

**Affiliations:** 1 Cardiff Metropolitan University, Cardiff, United Kingdom; 2 Manchester Metropolitan University, Manchester, United Kingdom; 3 Curtin University, Perth, Australia; 4 University of Birmingham, Birmingham, United Kingdom; University of Tartu, ESTONIA

## Abstract

Grounded in Duda’s integrated model of the motivational climate, the current study examined the hypothesized mediating role of motivation quality in the relationships between empowering and disempowering teacher-created motivational climates and indicators of quality engagement in secondary school physical education (PE). The hypothesised model was tested cross-sectionally and longitudinally in two separate samples of students. Data were collected via questionnaires measuring the motivational climate, autonomous and controlled motivation and indicators of engagement (enjoyment, concentration and boredom). Cross sectional data collected from 832 students (439 males and 386 females) while longitudinal data stemmed 299 students (166 males and 163 females). All students were from schools in Wales aged between 12 and 15 years. Structural equation modelling was used to test the hypothesised model and the mediating role of autonomous and controlled motivation. The hypothesised model was supported cross-sectionally and longitudinally, indicating that empowering climates positively predicted students’ autonomous motivation for PE, whereas disempowering motivational climates positively predicted controlled motivation. In turn, autonomous and controlled motivation positively and negatively predicted indictors of students’ engagement in PE in the hypothesised directions. Analyses revealed relationships between empowering and disempowering climates with enjoyment, concentration and boredom were indirect via autonomous and controlled motivation. In summary, results support the role of autonomous and controlled motivation in the differential relationships between empowering and disempowering motivational climates and indicators of the quality of student engagement. The findings suggest that targeted professional learning opportunities for PE teachers are needed which facilitate more empowering climates and reducing disempowering strategies.

## Introduction

Grounded in Duda’s [1] integrated model of the motivational climate, this study explored the motivation quality, the teacher created motivational climate and indicators of quality engagement in secondary school physical education (PE). Research has demonstrated that the teacher-created motivational climate plays a critical role in predicting variability in the quality of pupils’ motivation to engage in PE [2]. A considerable body of evidence supports the significance of the teacher-created motivational climate in PE for the prediction of differential cognitive, affective and behavioural outcomes for students [2,3]. The term, ‘motivational climate’ refers to the social psychological environment created by what significant others (such as PE teachers) say and do, how they provide feedback, and how they create the learning environment in lessons and school sports [2,4]. Building upon research conceptualising the motivational climate from a self-determination theory (SDT) [5,6] and achievement goal theory (AGT) [4,7] perspectives, Duda proposes that it is possible and important to simultaneously examine an interconnected array of facets of the social environment proposed by both theories. Specifically, based on the tenets of SDT and AGT and associated research evidence, Duda suggests the motivational climate can be more or less empowering and disempowering. Furthermore, pulling from the tenets of AGT and SDT, Duda’s [1] framework also suggests that empowering and disempowering climates will differentially predict motivation-related processes and indicators of optimal/compromised functioning and well/ill-being [see 8].

The present research aims to provide an initial test of Duda’s conceptual framework in secondary school PE. Specifically, across two studies, we examined the expected mediating role of students’ motivation regulations in the relationships between empowering and disempowering teacher-created motivational climates and indicators of quality engagement in Welsh secondary school PE.

### Empowering and disempowering motivational climates in PE

Adopting Duda’s model in PE is advantageous because, in contrast to previous research on the teacher-created motivational climate in PE that has generally been guided by AGT or SDT, it integrates and considers more comprehensively the array of the motivational climate dimensions which have pedagogical significance [9]. Based on the tenets of SDT, AGT and previous research, Duda [1] conceptualised empowering motivational climates as task-involving, autonomy-supportive and socially supportive. AGT proposes that a task-involved climate is fostered when the teacher values hard work, effort, skill development and students working together [4]. Within SDT, an autonomy-supportive climate is characterised by the teacher recognising students’ preferences, valuing meaningful choices, making decisions regarding learning and mastery that are student-centred, and ensuring a rationale is provided with requests [6]. SDT describes socially-supportive environments as those in which every student is important to the larger goals and objectives and feels valued and cared for as a person [10]. Duda [1] described a disempowering motivational climate as ego-involving and controlling. According to AGT, an ego-involved climate occurs when a teacher focuses attention on and rewards the best performing students and disapproves of student mistakes [4]. In contrast, SDT considers that a controlling climate pressures students to behave, think and feel in a specific way without acknowledging their perspectives [6,11].

Duda et al. [2] proposed that PE teachers who are more empowering will foster optimal functioning and engagement in PE students, whereas disempowering teaching strategies are more likely to be positively associated with maladaptive functioning and disengagement in PE. In support of these assumptions, previous research adopting either an SDT or AGT perspective has demonstrated a positive relationship between the dimensions underlying an empowering motivational climate and students’ enjoyment, concentrated effort and effective functioning [12–14], a greater focus on tasks, better peer relationships, and more significant effort and more persistence in PE classes [14,15]. Conversely, the particular characteristics of a disempowering motivational climate have emerged as a positive predictor of students’ amotivation, boredom and disengagement [16,17], reduced effort when failing, and diminished quality of relationships with others in PE lessons [18].

Although the aforementioned research has measured individual facets of the motivational climate proposed within Duda’s model in PE, until recently, no research has examined overall empowering and disempowering motivational climates created by teachers and their relevance for engagement-related outcomes in PE. This was partly due to there being no validated measure that captured empowering and disempowering motivational climates created by PE teachers. However, this measurement gap was addressed first by Milton and colleagues [9], who reported on the tailoring and subsequent validation of the Empowering and Disempowering Motivational Climate Questionnaire [EDMCQ, 19] for the PE context. The EDMCQ was initially developed within the youth sport context to capture adolescents’ perceptions of their coach’s use of empowering and disempowering strategies in training and competition. After contextualising the scale to PE, Milton et al. supported a two-factor structure that captured the two composite climate dimensions of empowering and disempowering. Subsequent studies have provided further support for the validity and reliability of students’ scores on Milton et al.’s adapted version of the EDMCQ [20]. More recently, involving a cross sectional sample of French secondary aged students, Mastagli, Van Hoye, Hainaut, and Bolmont [21] employed an alternative (and unpublished) measure of the perceived empowering (but not disempowering) PE teacher created motivational climate. The factor structure of the empowering scale was supported, and students’ scores on the scale were found to be reliable [21]. Moreover, empowering climates scores were positively related to students’ autonomy, competence, and relatedness need satisfaction, positive affect and reported concentration in PE.

### Autonomous and controlled motivation

In addition to highlighting the characterstics of empowering and disempowering motivational climates, Duda’s model draws from AGT and SDT to propose several psychological and motivational mechanisms (e.g., goal orientations, basic psychological needs, motivation regulations) that mediate the relationship between empowering and disempowering motivational climates in PE and differential indicators of students’ optimal/compromised functioning, degree of engagement and experiences of well-being/ill-being, respectively. One such mechanism is the extent to which students’ motivation for PE is more or less self-determined. SDT distinguishes between more autonomous and controlled motivations [5]; more autonomous forms of motivation represent participation that is regulated by interest and enjoyment or because of the personal value or understanding the importance of engaging. Controlled forms of motivation are when students feel pressure to participate either to protect their own perceived self-worth or external pressure from teachers, along with participating to avoid punishment and/or to gain praise and rewards [5,22].

Previous studies in sport have confirmed that adolescents who perceive the environment to be more empowering and/or less disempowering also report higher autonomous motivation [23,24]. Higher controlled motivation scores have been reported by young people who also perceive their psychological environment as more disempowering and/or less empowering [23,25,26]. Research in PE has also confirmed that students’ autonomous motivation predicts increased concentration, physical activity and greater engagement [22,27]. In comparison, motivations reflecting controlled reasons are generally positively correlated with adverse outcomes such as lower levels of physical activity, unhappiness and boredom [22,28]. These findings provide evidence of the inter-relationships between key constructs within Duda’s integrated model of the motivational climate and predicted consequences. To date, however, research has not directly tested the assumption that a young person’s quality of motivation for PE will mediate the relationship between their perceptions of empowering and disempowering motivational climates and indicators of quality of engagement. Moreover, studies which have examined the ‘motivational climate-motivation-outcomes’ mediation model in PE have tended to adopt a cross-sectional design [21,25] Longitudinal studies have been called for as they allow for the examination of changes in such variables over time and provide insight into cause-and-effect relationships [8,17,29]. This type of longitudinal study with more than one data point for all variables determines whether changes in one variable (e.g., empowering climate) predicts changes in a second variable (e.g., autonomous motivation) over time [30].

### The present study

**Fig 1:**
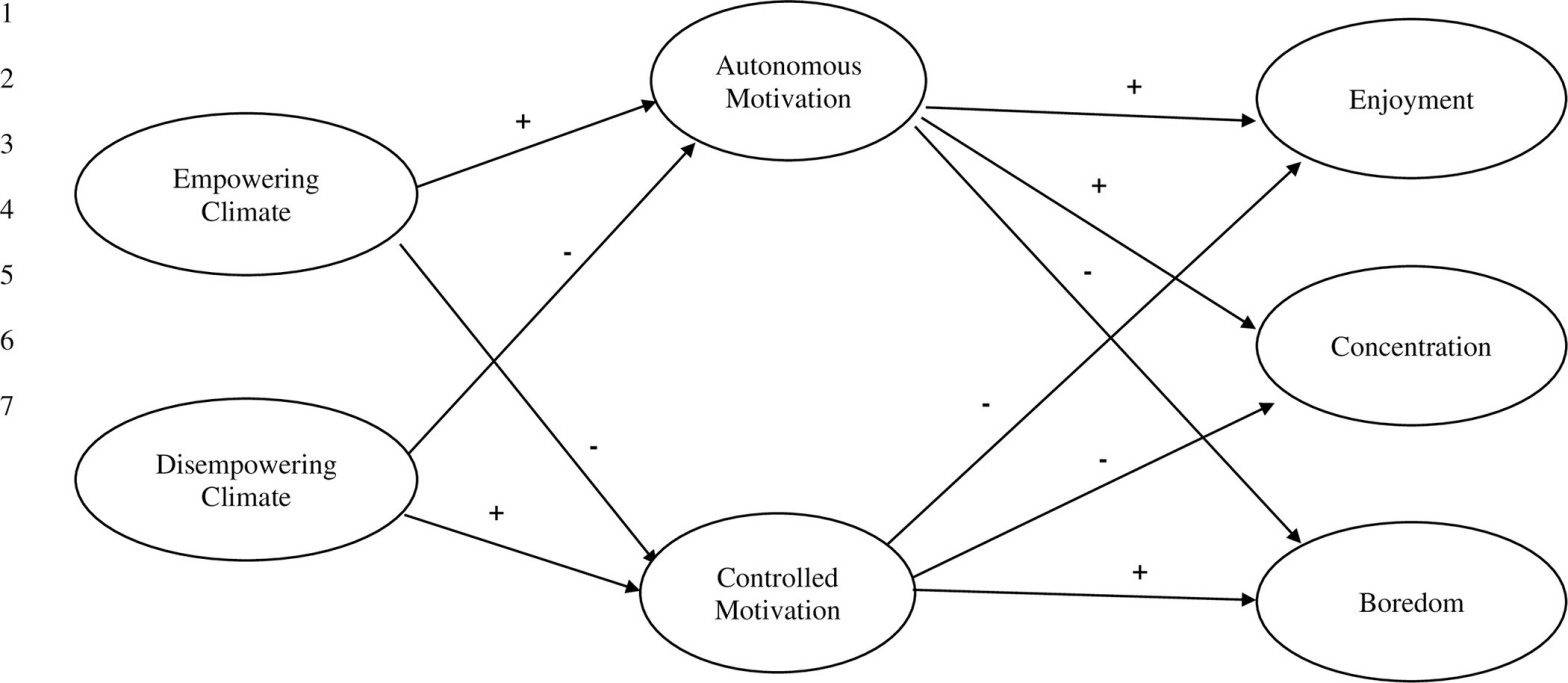
The role of motivation mediating the relationship between empowering/disempowering climates and indicators of quality engagement.

Drawing from Duda’s [1] integrated approach, the main aim of the present research was to examine the mediating role of motivation quality for PE (i.e., autonomous motivation, controlled motivation) in the relationships between empowering and disempowering features of the teacher-created motivational climate and indicators of quality engagement in PE (see Fig 1). The assumed mediational roles of autonomous and controlled motivation were tested cross-sectionally (study 1) and longitudinally (study 2) in Welsh secondary school students. Specifically, study one tested the hypothesised model at one time point during the school year across two samples of students with the aim of cross-validating the proposed model. In study two, we tested the model longitudinally over two-time points across the school year in a similar (albeit separate and different) sample of students recruited in study one.

## Study one

Study one provided an initial cross-sectional test of whether empowering and disempowering motivational climates were associated with variability in students’ engagement in PE (as reflected in reported enjoyment, concentration and boredom) via autonomous and controlled motivation. Based on the conceptual model proposed by Duda [1] and previous findings from youth sport [23,25,26], we hypothesised that students’ perceptions of an empowering climate would be positively associated with autonomous motivation and negatively with controlled motivation. In contrast, we expected that students’ perceptions of a disempowering climate would be negatively associated with autonomous motivation and positively with controlled motivation. Finally, we hypothesised that autonomous motivation would be positively associated with enjoyment and concentration and negatively correlated with boredom in PE. In contrast, the relationships were expected to be reversed for controlled motivation. We also hypothesised that autonomous and controlled motivation would mediate the associations between the climate dimensions and the targeted outcomes.

### Method

#### Participants

832 students (439 males and 386 females) from schools in South East Wales aged between 12 and 15 years old (M: 13.72; SD: 0.66) participated in this study.

#### Procedure

Ethics committees at the authors’ Universities (Birmingham University) approved the project. The first author subsequently made contact with headteachers to introduce the project. After receiving approval from headteachers, we arranged meetings with the PE department heads and teachers at each school. We provided a detailed overview of the study aims, procedures, and potential benefits for PE practice. Teachers had the opportunity to ask questions and voice any concerns. Written informed consent was obtained from all participating PE teachers. Parents were informed about the purposes of the research by letter and were allowed to withdraw their children from the project. Parents were provided with an information sheet and opt-out form. If parents did not wish their child to participate, they could return the signed opt-out form to the school or contact the researchers directly via email. A deadline was provided for opt-out responses.The project was also explained verbally and in writing to the students. Students who agreed to participate completed written consent forms and then completed a multi-section questionnaire.

Data collection took place at three secondary schools between Sep 2014 and 2015. The first author administered the questionnaires at the start of a lesson, and students responded without discussing answers with classmates or teachers. Attempts were made to minimise the teacher’s involvement by giving them a task to complete while the questionnaire was completed. The inventory took on average 20 minutes to complete. Students without parental consent or who chose not to participate were given an alternative educational task by their PE teacher (e.g. reading PE-related materials, completing a worksheet). This ensured all students were engaged in a productive activity during the data collection period.

The data were collected during the summer term, by which time the teachers had taught the students PE for 8 months.

#### Measures

All questionnaires were administered in English by the researcher. Participants responded to the items on a 5-point Likert scale ranging from 1 (strongly disagree) to 5 (strongly agree). When answering the questionnaires, the participants were instructed to “think about what it has usually been like in your PE lessons during the last 3–4 weeks”.

*Motivational climate*: Via the Empowering and Disempowering Motivational Climate Questionnaire-PE (EDMCQ-PE) [9], students’ perceptions of the teacher-created empowering (17 items) and disempowering (17 items) features of the motivational climate were assessed. The empowering climate items measure autonomy supportive (e.g. “My teacher gave students choices and options”), task involving (e.g. “My teacher made sure students felt successful when they improved”) and socially supportive (e.g. “My teacher could really be counted on to care, no matter what happened”) teaching strategies in PE. The disempowering climate items measure controlling (e.g. “My teacher yelled at students for messing up”) and ego-involving (e.g. “My teacher only praised students who performed best during a class”) teaching strategies in PE. Initial evidence supporting the psychometrics of the EDMCQ-PE were reported by Milton et al., [9].

*Motivation*: Students’ self-reported quality of motivation towards PE was measured using the perceived locus of causality questionnaire (PLOCQ) [31]. The items assess autonomous (e.g. “I take part in PE because PE is enjoyable) and controlled (e.g. “I take part in PE because that’s the rule”) motivation. Previous research supports the validity and reliability of secondary school aged students’ scores on the PLOCQ [32].

*Enjoyment*, *Boredom and Concentration*: Students’ self-reported enjoyment (e.g. “I usually had fun”) and boredom (e.g. “I usually wished the lesson would end quickly”) in PE lessons were measured using the subscales of the satisfaction interest scale (SIS) [33]. Students’ self-reported concentration in PE (e.g. “I thought carefully about the skills, tasks, and activities”) was measured via a scale developed by Standage et al. [28]. Young people’s scores on these measures have been reported to have acceptable reliability in previous research [28,33].

#### Data analysis

Data screening procedures were adopted to detect errors, outliers and normality in line with guidelines from [34]. Internal reliability was tested using Cronbach’s alpha. An alpha above .80 constitutes a reliable measure [35], while .70 and .60 are generally agreed as the lower limits for scales with >10 items or <10 items, respectively [36]. Descriptive analyses using SPSS were completed to generate subscale means and Pearson’s correlations, which examine the pattern of associations.

The first step when testing the hypothesised model involved adopting a parcelling approach. Parcelling was adopted to estimate the latent constructs of empowering and disempowering climates and autonomous and controlled motivation. As Little et al. [37] and Kline [38] described, parcelling has significant advantages when estimating multifaceted structural models: it is more stable, less parsimonious and less biased. A pragmatic approach to the use of parcelling was taken, and various methods of parcelling were considered. When properly constructed, parcels can clarify representations of even multidimensional constructs [39]. We followed Little et al’s [40] guidance on multidimensional parcelling. Firstly, any decisions must be theoretically justifiable and secondly, using three indicators per parcel, provide definitive tests of structural model parameters. In order to retain theoretical justification, we created parcels that represented each facet or characteristic of the construct under consideration, e.g., each empowering parcel contained items relating to autonomy support, social support and task involving climates [41]. This approach was consistently followed with disempowering and similarly autonomous and controlled motivation. The parcels retained their theoretical justification due to the few engagement indicators (enjoyment, concentration and boredom).

The hypothesised model was then tested using Structural Equation Modelling (SEM) based on maximum likelihood estimation samples in Mplus (Version 6.1) [41]. We did this across two samples. In each case, the model was tested using the robust maximum likelihood (MLR) estimator, which provides standard errors and fit indices that are robust to the Likert nature of the items and handling missing data. In order to test the stability and to cross-validate the hypothesised model, the data were split randomly in two within SPSS. Following the recommendations by Kline [38], the first stage was to check the fit of the overall measurement models. Goodness of fit was evaluated using the comparative fit index (CFI), the Tucker–Lewis index (TLI), and root mean square error of approximation (RMSEA) with 90% confidence intervals (CI). Hu and Bentler [42] proposed the following cutoff criteria: CFI and TLI >.90 and >.95 and RMSEA values < .08 and < .06, which are considered as indicators of acceptable and excellent fit, respectively. To allow a degree of flexibility in the cutoff criteria, the parameter estimates, statistical conformity and theoretical relevance were also consulted when evaluating and comparing model fit [43].

Thirdly, we tested the hypothesised model and the assumed mediating role of autonomous and controlled motivation using the MODEL INDIRECT command in Mplus as recommended by Cerin and Mackinnon [44]. Kelloway’s [45] procedures were used to test mediation where the researcher is encouraged to estimate the confidence interval (CI) around the indirect effect. There is evidence of mediation, or a specific indirect effect, when their 95% bootstrap-based confidence interval does not contain zero, i.e., zero is not included within the lower and upper bound Cis. Previous studies have investigated mediational models using the same approach [46]. Bootstrapping is a nonparametric resampling process that does not confirm the sampling distribution’s normality. Bootstrap-generated 95% bias-corrected confidence intervals (Cis) were constructed for 5000 samples on the hypothesised model [47].

### Results

#### Preliminary analyses

These were completed on the full sample of 832 students. Data screening procedures were adopted to detect outliers and normality in both samples in line with guidelines from Tabachnick and Fidell [34]. The internal consistency (see Table 1) estimates (α) for all the measures ranged from 0.75 to 0.91, indicating acceptable reliability. The mean scores (see Table 1) demonstrated that the sample perceived moderately high empowering climates and moderately low disempowering climates. Mean scores also showed relatively high levels of autonomous motivation, concentration and relatively low levels of controlled motivation and boredom (see Table 1).

**Table 1:** Internal consistency, means & correlations for cross sectional sample (n = 832).

Total Sample (832)	1	2	3	4	5	6	7	M	SD
1 Empowering	(.91)	-.54**	.55**	-.17**	.52**	.50**	-.41**	3.72	.62
2 Disempowering		(.86)	-.31**	.35**	-.32**	-.30**	.40**	2.72	.71
3 Autonomous Motivation			(.94)	-.13**	.83**	.69**	-.60**	3.87	.97
4 Controlled Motivation				(.75)	-.20**	-.11**	.43**	2.79	.77
5 Enjoyment					(.86)	.67**	-.67**	3.08	1.05
6 Concentration						(.89)	-.59**	3.74	.93
7 Boredom							(.84	2.39	1.08

**. Correlation is significant at the 0.01 level (2-tailed).

Internal Consistency scores in brackets ().

Bivariate correlations revealed that students’ perceptions of empowering climates were positively related with autonomous motivation, enjoyment and concentration and negatively correlated with controlled motivation and boredom. Disempowering climates were positively related with controlled motivation and boredom and negatively related with autonomous motivation, enjoyment and boredom. Consistent with Duda’s [1] framework, empowering and disempowering climates were negatively correlated. Following this, the sample was randomly split into two using SPSS before testing and cross validating the hypothesised model in Mplus.

#### Parcelling

Results for the parcelling approach revealed consistent and significant factor loadings for both samples with a range in random sample one from .17 to .90 and random sample two .18 and .91 (see Table 2).

**Table 2:** Parcelling approach: Factor loadings.

Parcels	Parcel 1	Parcel 2	Parcel 3
1 Empowering	.87** / .85**	.88** / .87**	.84** / .83**
2 Disempowering	.81** / .0.80**	.90** / .91**	.77** / .74**
3 Autonomous Motivation	.90** / .92**	.85** / .86**	.17** / .18**
4 Controlled Motivation	.35** / .28**	.90** / .86**	.50** / .85**

**. P-Value is significant at the 0.01 level (2-tailed).

Values to the left and right of the / left: Random sample 1 / right: Random sample 2

#### Assessment of model fit

The hypothesised models demonstrated an acceptable fit to the data across both random sample one (χ2 (12) = 383.18*; df = 175; CFI = .94; TLI = .93; RMSEA = .06; RMSEA 90%CI = .05 - .06) and random sample two (χ2 (12) = 655.67*; df = 175; CFI = .93; TLI = .92; RMSEA = .06; RMSEA 90%CI = .05 - .06) (* = p < .01).

#### Path analysis

**Fig 2:**
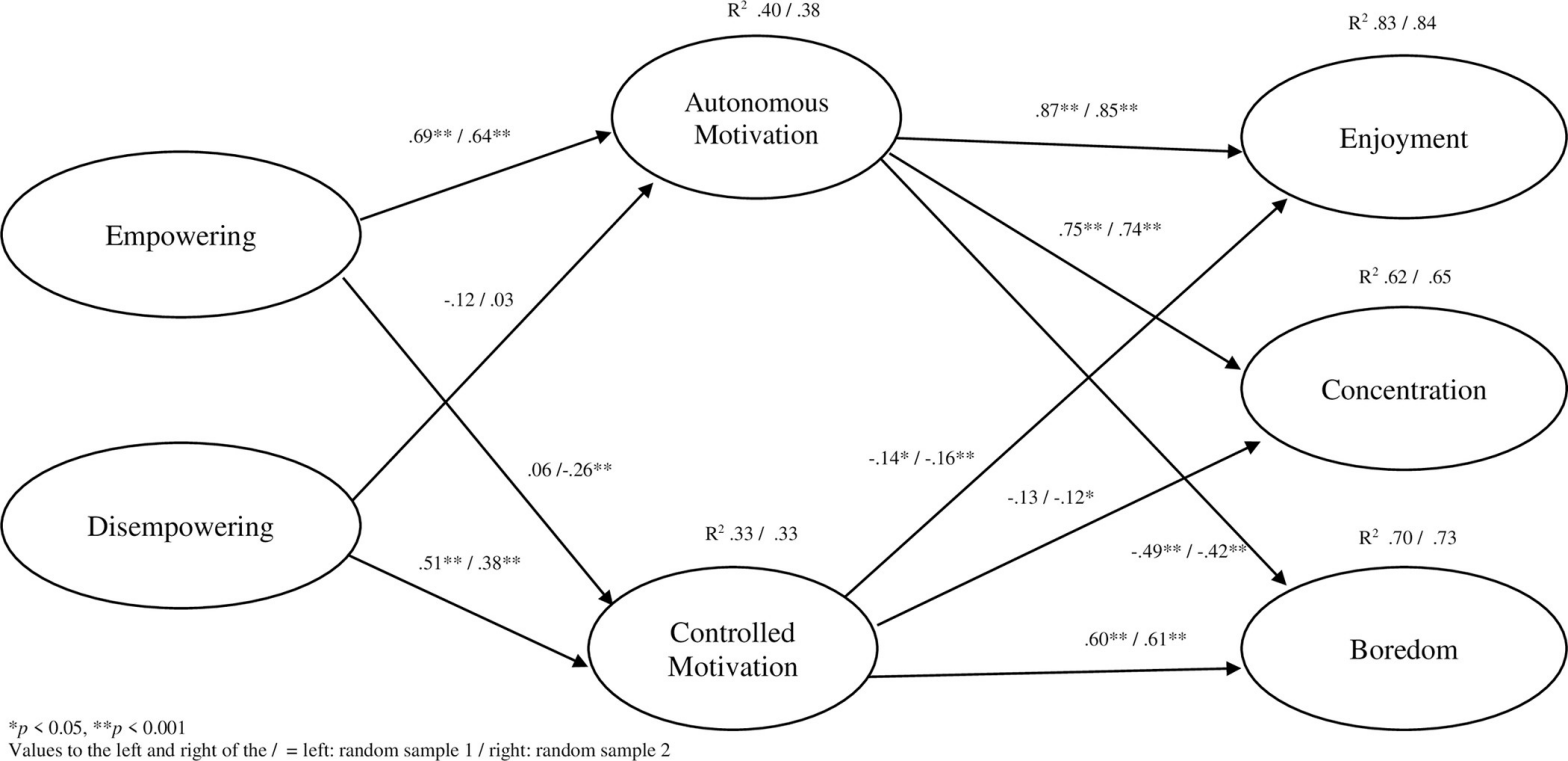
Cross sectional (Random 1 & 2) including estimates for the full model.

We sought to further explore the hypothesised associations between climate dimensions, motivation and outcomes (enjoyment, concentration and boredom). In random sample one (see Fig 2), perceptions of empowering climates were positively associated with autonomous motivation which in turn positively associated with enjoyment and concentration and negatively with boredom. Disempowering climates were positively associated with controlled motivation which in turn was positively associated with boredom and negatively correlated with concentration. These findings were replicated in random sample two (see Fig 2) with two exceptions, that is the observed negative associations between empowering climates and controlled motivation and controlled motivation and concentration. The effect sizes (R^2^) ranged from 0.33–0.84 in random sample one and 0.33–0.83 in random sample two (see Fig 2).

#### Indirect effects

The indirect effects and bootstrapped bias corrected 95% CI are reported in Table 3. Results in cross sectional sample one demonstrated that an empowering climate had a significant positive indirect effect on enjoyment and concentration, and a significant indirect effect on boredom, via autonomous motivation. In contrast, a disempowering climate had a significant positive indirect effect on boredom via controlled motivation and a small but significant negative indirect effect on enjoyment via controlled motivation. These relationships were replicated in cross sectional sample two with the exception of one additional indirect effect: a small but significant negative indirect effect between empowering and boredom via controlled motivation.

**Table 3:** Cross sectional (Random 1 & 2) indirect effects.

Indirect Effect			Random 1			Random 2		
Outcome	Motivation	Climate	Lower .5%	Estimate	Upper .5%	Lower .5%	Estimate	Upper .5%
**ENJOYMENT**	**Autonomous**	**Empowering**	*0*.*43*	*0*.61**	*0*.*76*	*0*.*40*	*0*.55**	*0*.*70*
	**Controlled**	**Empowering**	*-0*.*04*	***0*.*02***	*0*.*08*	*-0*.*00*	***0*.*04***	*0*.*08*
	**Autonomous**	**Disempowering**	*-0*.*09*	***0*.*05***	*0*.*19*	*-0*.*09*	***0*.*02***	*0*.*14*
	**Controlled**	**Disempowering**	*-0*.*14*	*-0*.07*	*-0*.*02*	*-0*.*10*	*-0*.06*	*-0*.*02*
**BOREDOM**	**Autonomous**	**Empowering**	*-0*.*50*	*-0*.34**	*-0*.*17*	*-0*.*38*	*-0*.27**	*-0*.*16*
	**Controlled**	**Empowering**	*-0*.*28*	***-0*.*07***	*0*.*14*	*-0*.*27*	*-0*.16**	*-0*.*04*
	**Autonomous**	**Disempowering**	*-0*.*11*	***0*.*02***	*0*.*06*	*-0*.*07*	***-0*.*01***	*0*.*05*
	**Controlled**	**Disempowering**	*0*.*11*	*0*.30**	*0*.*49*	*0*.*10*	*0*.23**	*0*.*36*
**CONCENTRATION**	**Autonomous**	**Empowering**	*0*.*34*	*0*.52**	*0*.*70*	*0*.*33*	*0*.47**	*0*.*62*
	**Controlled**	**Empowering**	*-0*.*04*	***0*.*02***	*0*.*07*	*-0*.*01*	***0*.*03***	*0*.*07*
	**Autonomous**	**Disempowering**	*-0*.*09*	***0*.*04***	*0*.*17*	*-0*.*08*	***0*.*02***	*0*.*12*
	**Controlled**	**Disempowering**	*-0*.*17*	***-0*.*07***	*0*.*03*	*-0*.*10*	***-0*.*05***	*0*.*01*

## Study 2

The findings of Study 1 highlighted that the cross-sectional relationships between empowering and disempowering motivational climates with indicators of students’ engagement (enjoyment, concentration and boredom) are mediated by the quality of their motivation in PE. Therefore, the first objective of Study 2 was to use an independent sample to test the hypothesised model longitudinally over two time points within the school year.

### Procedure

The recruitment procedure replicated that of study one. Data collection took place in a total of 11 secondary schools across Wales during the same time period. A small team (5 in total) of trained data collectors led by the first author administered the questionnaires, and students completed the inventory without discussing answers with classmates or teachers. One school completed an online version of the questionnaire administered using the same procedures (albeit electronically). The students completed the questionnaire at timepoint one (T1) in October and November, with timepoint two (T2) in February and March. Due to school-related issues, one school dropped out of the study at time two. We matched the students’ responses over the two-time points by creating an anonymised coding system.

### Participants

534 students (272 males and 262 females; SD: 0.72) from Years 8 to 10 completed the questionnaire at T1 and 299 students (166 males and 163 females; SD: 0.64) completed the questionnaire at the T2. Students were recruited from schools across all regions in Wales and took PE classes as part of their weekly school curriculum.

### Measures

The measures used were the same as in study one.

### Data analysis

The statistical analyses were performed using the following steps. As per study one, internal reliability and descriptive analyses were produced in SPSS. Next, a one-way between-group analysis of variance was conducted to compare the scores for those students who completed the T1 questionnaire versus students who completed T1 and T2 questionnaires. The students who only completed T1 were subsequently removed. The same parcelling approach was taken as per study one. A confirmatory factor analysis (CFA) was performed to assess the suitability of the proposed measurement model and to estimate error-free correlations between the latent variables. Assessment of model fit followed the same procedures as study one.

We formed a structural equation path model to test the relationship between the variables at the two time points, and the path analysis at T2 to answer the specific research questions. In order to test the equivalence of factor loadings over time following the CFA, we enforced the requisite equality constraints, constraining comparable parameters from each time wave using the BY command within Mplus [48]. We examined possible mediation (indirect effects) between variables in line with the hypotheses. The hypothesized model focused on the variables at T2, and therefore the indirect effects only included the paths between the T2 variables. The parcelling, bootstrapping, and mediation approach was followed using the same procedures as study one.

### Results

#### Preliminary analyses

The percentage of missing data at T2 (41%) was partly due to a school that dropped out of the study after T1 data collection (21% of the missing data). The rest of the missing data were from students in attendance at T1 but absent at T2 across the schools. Results of ANOVAs revealed there was a statistically significant difference for disempowering, F (1, 534) = 4.96 p = 0.03, autonomous motivation, F (1, 533) = 4.33 p = 0.04, concentration, F (1, 530) = 5.38 p = 0.02 and boredom, F (1, 531) = 5.63 p = 0.02 between the two groups (those in attendance and those absent). Despite reaching statistical significance, the actual difference in mean scores between the groups (see Table 4) was minimal, with the effect size calculated using eta squared being smaller than .01 for all four variables. There was no statistically significant difference for empowering, controlled motivation and enjoyment. Even so, the differential attrition is considered as a limitation to the generalizability of the results and discussed further below. The listwise deletion of the missing participants was chosen because the data were not missing at random.

**Table 4:** Means, standard deviation, internal consistency and correlations for longitudinal sample.

Total Sample at T1 & T2 (299)	1	2	3	4	5	6	7	8	9	10	11	12	13	14	M	SD
1 Empowering T1	(.90)	.60**	-.54**	-.35**	.51**	.38**	-.23**	-.13*	.46**	.41**	.46**	.40**	-.48**	-.30**	3.72	.62
2 Empowering T2		(.90)	-.43**	-.51**	.42**	.52**	-.19**	-.20**	.35**	.53**	.34**	.45**	-.41**	-.43**	3.68	.62
3 Disempowering T1			(.86)	.67**	-.21**	-.18**	.35**	.17**	-.16**	-.24**	-.20**	-.20**	.35**	.24**	2.72	.71
4 Disempowering T2				.85)	-.13**	-.23**	.26**	.32**	-.11	-.23**	-.13*	-.18**	.22**	.32**	2.80	.71
5 Autonomous Motivation T1					(.88)	.69**	-.20**	-.14*	.78**	.57**	.63**	.53**	-.66*	-.55**	3.87	.97
6 Autonomous Motivation T2						(.90)	-.17**	-.07	.56**	.77**	.48**	.66**	-.55**	-.65**	3.90	.88
7 Controlled Motivation T1							(.67)	.40**	-.18**	-.17**	-.18**	-.20**	.39**	.29**	2.79	.77
8 Controlled Motivation T2								(.64)	-.15*	.09	-.09	-.05	.21**	.31**	2.88	.75
9 Enjoyment T1									(.89)	.55**	.63**	.44**	-.64**	.47**	3.08	1.05
10 Enjoyment T2										(.90)	.43**	.63**	-.52**	-.67**	3.91	.96
11 Concentration T1											(.81)	.55**	-.61**	-.50**	3.74	.93
12 Concentration T2												(.82)	-.40**	-.58**	3.73	.92
13 Boredom T1													(.86)	.55**	2.39	1.08
14 Boredom T2														(.87)	2.21	.99

**. Correlation is significant at the 0.01 level (2-tailed).

*. Correlation is significant at the 0.05 level (2-tailed).

Descriptive statistics and internal reliability tests were then completed on the sample of 299 students who completed the questionnaire at both time points. The mean scores (see Table 4) demonstrated that the sample perceived moderately high empowering climates and moderately low disempowering climates. Mean scores from both T1 and T2 showed relatively high means for autonomous motivation, concentration and relatively low controlled motivation and boredom. At T2, there was a higher mean for enjoyment than T1 enjoyment (see Table 4). Bivariate correlations (see Table 4) revealed across both T1 and T2 that students’ perceptions of empowering climates were positively related to autonomous motivation, enjoyment and concentration and negatively related to controlled motivation and boredom. Across both T1 and T2, disempowering climates were positively related with controlled motivation and boredom and negatively related with autonomous motivation, enjoyment and concentration.

#### Assessment of model fit

Results for the parcelling approach revealed consistent and significant factor loadings ranging from .52 and .92. Model fit from the hypothesised model including the relationships between the variables at T1 and T2 demonstrated an acceptable fit to the data 1: χ2 (12) = 638.38*; df = 372; CFI = .94; TLI = .93; RMSEA = .05 CI: 0.04–0.06); (* = p < .01).

#### Longitudinal test of model

**Fig 3:**
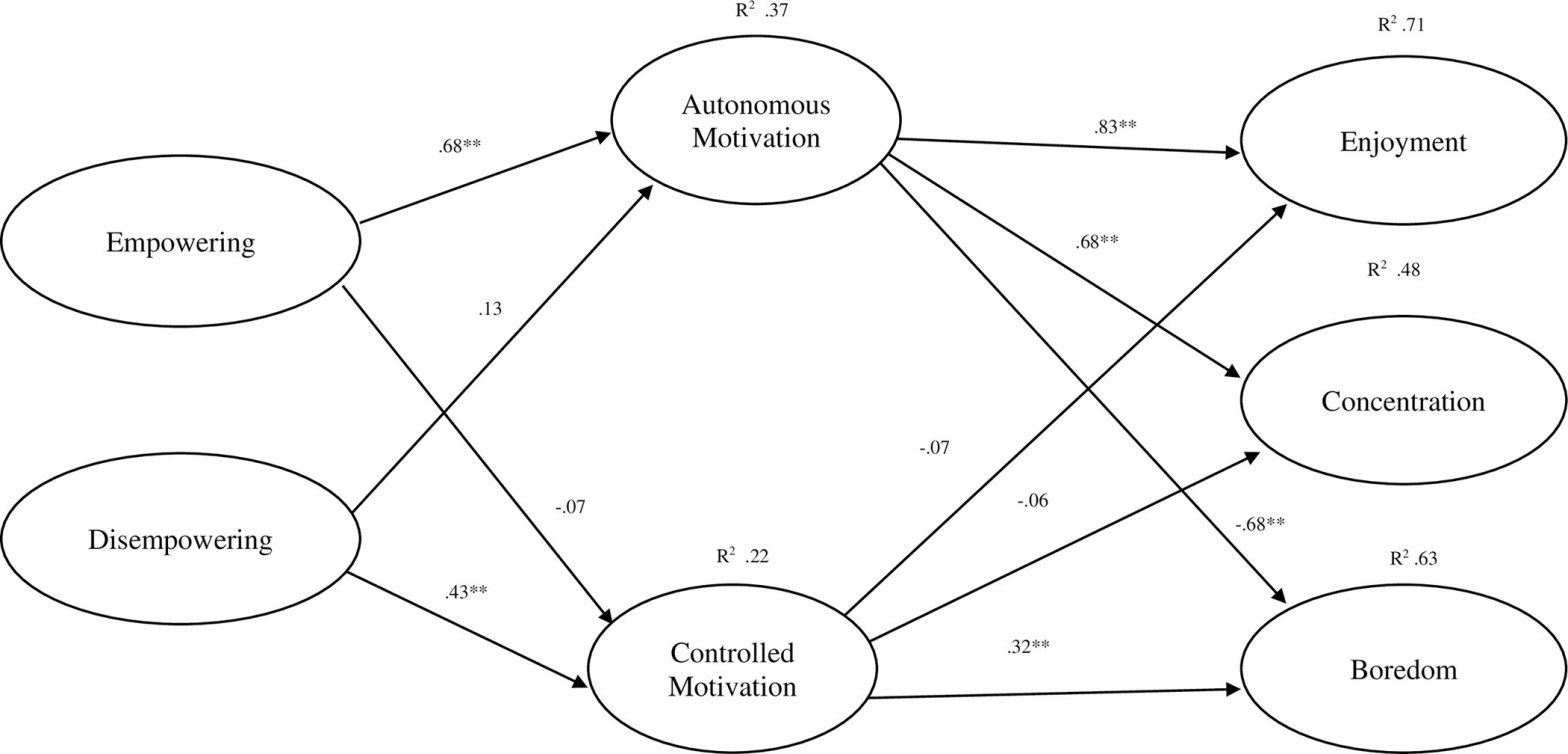
Longitudinal estimates for the full model at timepoint 2 (controlling for scores at time one).

Parameter estimates for the relationships between each variable at T1 and T2 showed significant strong positive relationships. The correlations between each variable across T1 and T2 ranged between λ| = .41 to .72 (see Table 5). Concerning the parameter estimates of the proposed model at time point 2 controlling for T1 scores (see Fig 3), perceptions of empowering climates were positively associated with autonomous motivation (λ| = .68) which in turn was strongly associated with enjoyment (λ| = .83), concentration (λ| = .68) and negatively associated with boredom (λ| = -.68). Disempowering climates were positively associated with controlled motivation (λ| = .43), which were positively associated with boredom (λ| = .32). The effect sizes (R^2^) ranged from 0.22–0.71 (see Fig 3).

**Table 5:** Paramter estimates showing the relationship between variables at Time 1 and Time 2.

Total Sample at T1 & T2 (299)	Paramter Estimate
1 Empowering T1 & T2	.60**
2 Disempowering T1 & T2	.72**
3 Autonomous Motivation T1 & T2	.68**
4 Autonomous Motivation T1 & T2	.51**
5 Enjoyment T1 & T2	.43**
6 Concentration T1 & T2	.41**
7 Boredom T1 & T2	.45**

**. Correlation is significant at the 0.01 level (2-tailed).

#### Indirect effects

The indirect effects and bootstrapped bias-corrected 95% CI’s are reported in Table 6. Results demonstrated that empowering climate scores had a significant positive indirect relationship with enjoyment and concentration, and a negative indirect relationship with boredom, via its positive association with autonomous motivation. In contrast, disempowering climate scores had a significant positive indirect relationship with boredom via controlled motivation. No other significant indirect effects were observed.

**Table 6:** Longitudinal indirect effects.

Indirect Effect			Lower .5%	Estimate	Upper .5%
Outcome	Motivatrion	Climate			
**ENJOYMENT**	**Autonomous**	**Empowering**	*0*.*66*	*1*.01**	*1*.*43*
	**Controlled**	**Empowering**	*-0*.*02*	***0*.*01***	*0*.*10*
	**Autonomous**	**Disempowering**	*-0*.*04*	***0*.*15***	*0*.*36*
	**Controlled**	**Disempowering**	*-0*.*13*	***-0*.*04***	*0*.*01*
**BOREDOM**	**Autonomous**	**Empowering**	*-1*.*21*	*-0*.85**	*-0*.*56*
	**Controlled**	**Empowering**	*-0*.*22*	***-0*.*04***	*0*.*13*
	**Autonomous**	**Disempowering**	*-0*.*30*	***-0*.*12***	*0*.*03*
	**Controlled**	**Disempowering**	*0*.*08*	*0*.20**	*0*.*38*
**CONCENTRATION**	**Autonomous**	**Empowering**	*0*.*49*	*0*.78**	1.15
	**Controlled**	**Empowering**	*-0*.*02*	***0*.*01***	*0*.*11*
	**Autonomous**	**Disempowering**	*-0*.*03*	***0*.*11***	*0*.*28*
	**Controlled**	**Disempowering**	*-0*.*13*	***-0*.*03***	*0*.*04*

## Discussion

The primary purpose of the present research was to provide an initial test of Duda’s [1] conceptual model of the motivational climate in Welsh secondary school PE. Duda’s model posits that empowering motivational climates in PE facilitate the quality of students’ engagement, whereas disempowering motivational climates promotes disengagement (boredom) through the climate dimensions’ differential relationships with autonomous and controlled motivation. Specifically, the model posits that empowering climates positively predict students’ autonomous motivation for PE, whereas disempowering motivational climates positively predict controlled motivation. Finally, autonomous and controlled motivation are expected to positively and negatively predict the quality of students’ engagement in PE, respectively. This model was supported in two studies, including a cross sectional and a longitudinal 6-month prospective study, with secondary school students from Wales.

### The role of empowering and disempo”erin’ motivational climates in students’ engagement in PE

The present findings underscore the extent to which teacher-created empowering and disempowering motivational climates matter in regard to the quality of students’ engagement in PE. Adolescents may experience a range of physical, psychological, social and emotional benefits in PE when fully and optimally engaged [49]. The findings from this study suggest that such engagement may depend on the PE teacher creating empowering motivational climates and reducing disempowering strategies. Cross-sectional and longitudinal bivariate correlations confirmed that empowering motivational climates were positively associated with students’ enjoyment and concentration and negatively associated with boredom in PE. In contrast, disempowering motivational climates were positively associated with students’ self-reported boredom and negatively correlated with enjoyment and concentration in PE. These findings are in line with previous research in PE, which has shown that individual facets of empowering climates (e.g., task-involving or autonomy support or socially support) are positively related to indicators of students’ quality engagement in PE. In contrast, specific characteristics of a disempowering teacher-created climate (i.e., ego-involving or controlling) negatively correlate to students’ PE participation [12–14]. The current findings align with those of Mastagli et al. [21], who reported a positive association between students’ perceptions of empowering teacher-created motivational climates and their level of concentration when participating in PE classes. The present findings are also in accord with past research that has tested Duda’s [1] conceptual model in youth sport, which has shown that empowering motivational climates predict adaptive outcomes. On the other hand, disempowering climates typically leads to less adaptive and more maladaptive outcomes [8,25,50].

### The mediating role of autonomous and controlled motivation

The present findings also offer initial support for aspects of Duda’s and colleagues’ [8] conceptual model by highlighting the critical mediational role of a students’ motives in the relationships between empowering and disempowering climates and indicators of engagement in PE. Across both studies, autonomous motivation for PE mediated the positive relationships between the teacher-created empowering motivational climate and indicators of students’ engagement (i.e., enjoyment and concentration). As such, our findings on the role of autonomous motivation as a mediator of the effect of empowering motivational climates parallel previous studies conducted in youth sport [23,25]. Study one also suggests that students’ controlled motivation may be a mediator in the negative relationship between empowering climates and students’ reported experiences of boredom in PE. However, this particular finding was limited to sample two in study one and was not replicated longitudinally. Thus, future research is needed to determine whether this particular finding is novel to a particular sample or is reproducible.

In contrast, controlled motivation for PE mediated the positive relationships between perceptions of the teacher-created disempowering motivational climate and boredom, and in study one, the negative cross-sectional relationship with enjoyment. Disempowering motivational climates, characterised by punitive, controlling teaching strategies that emphasise comparative ability, lead students to engage in PE for guilt, recognition seeking and the avoidance of disapproval/demonstration of low ability.

Based on the critical assumptions of SDT and AGT, Duda’s [1] model offers a potential explanation for the mediating role of students’ autonomous and controlled motivation on the effects of empowering and disempowering teacher-created motivational climates in PE. According to the model, empowering teaching strategies are more likely to foster autonomous motives towards PE (and fewer controlled motives) via the satisfaction of students’ psychological needs of autonomy, relatedness and competence. Moreover, Duda’s model proposes that an empowering motivational climate is more likely to encourage students to view competence in a task-involving manner (as defined in AGT). In contrast, disempowering strategies are more likely to foster controlled motives towards PE (and possibly fewer autonomous motives) via the dissatisfaction and/or frustration of students’ psychological needs and the promotion of an ego-involving view of competence (as defined in AGT). Support for mediating role of basic psychological needs in the relationship between empowering and disempowering motivational climates and self-determined motivation was recently provided in the youth sport context [25]. To date, the mediating role of basic psychological needs (and views of competence specifically) in the relationship between empowering and disempowering motivational climates and students’ motivation in PE remained unexplored. Thus, future research may wish to address this gap in the literature.

### Limitations and future directions

Although this study has several noteworthy strengths (i.e., multiple samples; cross-sectional and longitudinal designs), there are limitations for consideration. Attrition is a common issue in longitudinal research as mentioned in the results of study two, 41% of the participants did not complete the questionnaire at Time 2. This attrition was due to a mixture of one school dropping out of the study and student absences alongside another school changing their groupings due to timetable changes between the timepoints. In addition, there were significant differences in scores on the targeted variables for dropouts compared to students who participated at both time points, albeit these differences were small. Future research seeking to re-test the relationships outlined in this paper should limit the loss of generalizability caused by nonrandom attrition with multiple waves of data collection [51]. There could also be further strategies used to mitigate attrition such as scheduling follow-ups to collect data from absent students, providing appropriate incentives for continued participation, maintaining better contact with participants between time points and oversampling at baseline to account for expected attrition.

According to Stenling et al., [30], whilst it is possible to test relationships longitudinally with data from two-time points, this limits one’s ability to model non-linear forms of change and unravel actual change from measurement error [52]. Therefore, future studies should look to collect data on the targeted variables in this study over three or more time points [53]. Future studies could also use samples with diversified subgroups of students to assess the invariance of the proposed measurement and structural models across, for example, gender, age groups, and students from different countries. Finally, this study was also limited by the exclusive reliance on self-report measures, and future research may wish to obtain self-report and observational ratings of the targeted variables when and where possible via validated scale. For example, researchers may wish to adapt the Multidimensional Motivational Climate Objective System [24] to rate the extent to which the motivational climate in PE is more or less empowering and disempowering [1] and the Engagement Rating Scale as an objective measure of students’ engagement in PE [54].

## Conclusion

The present study provides robust empirical evidence supporting the mediating roles of autonomous and controlled motivation in the relationships between motivational climates and students’ engagement quality in physical education (PE). This research aligns with and extends Duda’s [1] conceptual model, demonstrating how variations in motivational climates distinctly predict optimal engagement in specific activities. While the findings substantiate key aspects of the model, further investigation is required to address inconsistencies observed between cross-sectional and longitudinal studies. The originality of this research lies in its dual approach, integrating both cross-sectional and longitudinal data to offer a comprehensive understanding of motivational dynamics in PE settings. The methodological rigour is evident in the thorough analysis and the use of validated instruments to assess motivational climates and engagement indicators. These findings have significant implications, emphasizing the critical role of professional development for PE teachers. Specifically, the research underscores the need for training programs focused on fostering empowering climates and minimizing disempowering practices to enhance student motivation and engagement in PE. Such practical applications are vital for optimising educational outcomes and advancing pedagogical practices in physical education [20,55].
